# Evaluating a Speech-Specific and a Computerized Step-Training-Specific Rhythmic Intervention in Parkinson's Disease: A Cross-Over, Multi-Arms Parallel Study

**DOI:** 10.3389/fresc.2021.783259

**Published:** 2022-01-14

**Authors:** Anne Dorothée Rösch, Ethan Taub, Ute Gschwandtner, Peter Fuhr

**Affiliations:** ^1^Department of Clinical Neurophysiology/Neurology, Hospital of the University of Basel, Basel, Switzerland; ^2^Department of Neurosurgery, Hospital of the University of Basel, Basel, Switzerland

**Keywords:** Parkinson's Disease, rhythmic deficits, rhythmic interventions, balance-mobility step-training, rhythmic speech-language therapy

## Abstract

**Background:**

Recent studies suggest movements of speech and gait in patients with Parkinson's Disease (PD) are impaired by a common underlying rhythmic dysfunction. If this being the case, motor deficits in speech and gait should equally benefit from rhythmic interventions regardless of whether it is a speech-specific or step-training-specific approach.

**Objective:**

In this intervention trial, we studied the effects of two rhythmic interventions on speech and gait. These rhythmic intervention programs are similar in terms of intensity and frequency (i.e., 3x per week, 45 min-long sessions for 4 weeks in total), but differ regarding therapeutic approach (rhythmic speech vs. rhythmic balance-mobility training).

**Methods:**

This study is a cross-over, parallel multi-arms, single blind intervention trial, in which PD patients treated with rhythmic speech-language therapy (rSLT; *N* = 16), rhythmic balance-mobility training (rBMT; *N* = 10), or no therapy (NT; *N* = 18) were compared to healthy controls (HC; *N* = 17; matched by age, sex, and education: *p* > 0.82). Velocity and cadence in speech and gait were evaluated at baseline (BL), 4 weeks (4W-T1), and 6 months (6M-T2) and correlated.

**Results:**

Parameters in speech and gait (i.e., speaking and walking velocity, as well as speech rhythm with gait cadence) were positively correlated across groups (*p* < 0.01). Statistical analyses involved *repeated measures* ANOVA across groups and time, as well as *independent* and *one-samples t-tests* for within groups analyses. Statistical analyses were amplified using *Reliable Change (RC)* and *Reliable Change Indexes (RCI)* to calculate true clinically significant changes due to the treatment on a patient individual level. Rhythmic intervention groups improved across variables and time (total Mean Difference: 3.07 [SD 1.8]; 95% CI 0.2–11.36]) compared to the NT group, whose performance declined significantly at 6 months (*p* < 0.01). HC outperformed rBMT and NT groups across variables and time (*p* < 0.001); the rSLT performed similarly to HC at 4 weeks and 6 months in speech rhythm and respiration.

**Conclusions:**

Speech and gait deficits in PD may share a common mechanism in the underlying cortical circuits. Further, rSLT was more beneficial to dysrhythmic PD patients than rBMT, likely because of the nature of the rhythmic cue.

## Introduction

Parkinson's Disease (PD) is a neurodegenerative disease related to loss of dopaminergic neurons in the substantia nigra (1). The dopaminergic deficiency impairs the functioning of cortical circuits in the basal ganglia (BG) (2). The BG, premotor cortex, supplementary motor area (SMA), and the cerebellum are responsible for the smooth regulation of sequential motor functions (3) including speech articulation, postural stability, and locomotion (4).

Movements necessary for speaking and walking require constant and exact tempo-spatial sequencing of antagonistic muscle pairs as well as discrete error correction. In more detail, neuroimaging examinations of healthy motor action showed the BG and SMA to build a functional loop for sequential movement preparation, which then switches into a “readiness state” for forthcoming predictable movements (5, 6). Upon movement initiation, this cortical loop changes into automated sequential neuron discharges after every sub-movement (7), and the initial loop is expanded to further cortical parts [i.e., motor cortex and the cerebellum; (6)]. Yet, neurodegenerative diseases impair these cortical loops, and PD patients would then have to pay conscious attention to the performance of movements (e.g., when walking or speaking) that would otherwise be performed with ease, leading to an increased cognitive demand and to slowed reaction times (3, 8).

### Parkinsonian Symptoms in Gait and Speech

Gait impairment in PD can manifest itself with slowness of walking and disrupted cadence due to reduced step length (9, 10), postural instability (11), freezing of gait (FOG) (12, 13), or sway (14–16).

Speech impairments in PD are termed dysarthria and may further be categorized into seven conditions (17–19): ataxic, flaccid, spastic, (rigid-)hypokinetic, hyperkinetic, mixed, unilateral upper motor neuron. Furthermore, recent research suggests an early type of dysarthria with pronounced deficiencies in speech rhythm, prosody and intonation (20–23), that is a dysarthria at an early-stage of PD before speech deficits further progress and may be attributed to a specific type of dysarthria (21–23).

Importantly, these dysarthria types differ in terms of what subsystems (i.e., respiratory, articulatory, phonatory, and prosody) are affected and to what degree. Although some types of dysarthria cause a reduction of speech rate (17, 24–29), others result in an acceleration (27, 30–35) or preservation (33, 36, 37) of it. Yet, most importantly is the type of underlying deficit causing the speech unintelligibility and the loss of naturalness of speech: While some dysarthrias benefit from an amplitude-orientated approach [i.e., targeting loudness with combined variations in pitch; (38, 39)], other dysarthrias seem to have an impaired speech rhythm and timing-deficit (20, 33, 40). As a fact, only a therapeutic approach targeting successfully the underlying deficit, will further improve motor articulation, respiration, phonation and finally, speech intelligibility.

Notably, this study here focused on PD patients with dysrhythmic types of dysarthria, i.e., early-stage dysarthria, (rigid-)hypokinetic, and ataxic dysarthria. Speech impairment in dysrhythmic PD is due to rigid, hypokinetic or bradykinetic articulation and asynchronous-asymmetrical breathing, leading to deformed speech with a reduced variability in accentuations, slurred motor articulation (39, 41) with non-physiologic pauses [i.e., quantitatively more speaking pauses with inadequate breathing; (24)] and suddenly occurring rushed speaking sequences (17, 19, 33, 40, 42).

Early research suggested that dysrhythmic speech in PD develops in line with spatiotemporal gait disorders, as motor articulation and speech rate decreases similarly to walking velocity (43–46), and the inter-pause speech duration (ISD) shortens similarly to stride or step length (43, 47, 48), and also that paradoxical speech rhythms may be linked to gait festination and freezing (43, 48–50). Therefore, a common underlying dysrhythmic deficit in gait and speech in PD has been assumed and the term “general dysrhythmia” has been introduced (20, 40, 48, 50–53).

### Treatments in PD

As for treatment, recent findings imply that speech dysfluency cannot be improved to the same extent as gait is improved in PD patients by deep brain stimulation in the subthalamic nucleus (DBS-STN) (43, 54, 55). As for levodopa treatment, the medication showed robust improvements in gait, whereas mixed results were reported for speech impairments (54–57).

Turning to rhythmic auditory stimulation (RAS) (58, 59) or specific dancing for PD (5, 9, 52, 53, 60–65), these interventions are types of neurologic rhythmic therapy and have shown beneficial effects in terms of increasing step length, reducing falls and freezing of gait. While RAS may be on the basis of a simple metronome beat and range up to more complex music with highlighted pulses, dancing may involve traditional partner-dancing [Argentine tango dancing (66)] or virtual single-dance step-trainings (9, 52). Turning to rhythmic speech methods, these may involve singing, vocal intonation therapy (VIT) or rhythmic speech cueing (RSC) (18, 50). Although these rhythmic interventions vary in terms of frequency, intensity, methods, and whether external devices are needed, all rhythmic interventions intend to re-activate intrinsically rhythmical movements in order to facilitate movement initiation and execution (58, 59).

*How does rhythm influence motor action?* As a fact, cortical structures such as the BG (58, 60, 67–78), cerebellum, premotor cortex and SMA subserving movement initiation, execution and monitoring, were also reported responsible for the perception and production of rhythm (with or without involved motor action) (69, 72, 79–84). Neuroimaging studies showed neural coupling activity between sensory-auditory and premotor cortex during rhythm processing in healthy populations (69, 72, 85) and in PD patients (86, 87). These neural couplings build the fundamentals of *rhythmic entrainment*, where auditory cues may drive motor action (88).

*Rhythmic entrainment* may be triggered in PD patients in gait (64, 65) and speech (89, 90). Thus, although patients with PD experience deficiencies with movement initiation and control, their response to rhythmic cues remains intact throughout disease progression (5, 40, 87). In other words, motor deficiencies may be compensated via *rhythmic cues*. Yet, to-date no study has investigated effects of rhythmic cuing on both speech and gait.

*Why may dancing improve speech?* Dancing was reported to improve heart rate, breathing, muscular strength and coordination (91–93). Thus, when dancing the human body has to build up and constantly maintain a physically balanced muscle tonus throughout the whole body in order to move to the rhythm, sometimes hold positions and maintain postural stability. By that, this whole body muscle tone goes from head to toe, including facial and articulation muscles, e.g., the tongue and velum, as well as the diaphragm for breathing control. Therefore hypothetically, dancing may also affect motor articulation muscles as a cross-over effect. However, such cross-over effects remain uninvestigated to-date.

*Why may rhythmic speech therapy have an effect on gait?* Singing was reported to have a beneficial effect on walking cadence and velocity (94, 95). This may be because singing places relatively low cognitive demands and may be easily performed during walking. Further, it can easily be adapted to individual's tempi (94, 95). As thus, internal singing is a robust cue to activate walking and maintain a comfortable pace via the concept of rhythmic entrainment (94, 95). Yet, the feasibility of internal singing while every day communication (e.g., ordering a bread at the bakery) may be cognitively too challenging (96). The reason is that both language modes (i.e., singing and spontaneous speech production) employ an activation of the same cortical areas responsible for language production, but target different output modes. Thus, these two would compete at the same level of cortical activation making inhibition control at the level of output mode impossible. On the contrary, a metered and patterned rhythmic cueing method led to improved speech abilities in PD patients (97). Still, possible effects of a rhythmic speech approach on gait remain uninvestigated to-date.

Therefore, the question arises whether a rhythmic step-training or a rhythmic speech intervention might have crossover effects on speech or gait parameters, respectively. This would lend support to the hypothesis that gait and speech disturbances are somewhat related by possibly sharing a common rhythmic foundation and could therefore benefit from a rhythmic intervention regardless of its primary focus.

### Study Aim

In this crossover study, we compared two rhythmic interventions (i.e., a specific type of rhythmic speech-language therapy vs. a rhythmic virtual step training) in PD in order to investigate their effects (and crossover effects) on deficiencies in cadence and velocity in speech and gait. We also compared the results to findings in an untreated control group of PD patients (“the waiting list”) and in untreated healthy controls. We did this to determine whether a dysrhythmic deficit affects speech and gait in a similar way, and whether dysrhythmic movements in speech and gait might benefit from rhythmic interventions. Thus, we formulated the following hypotheses:

*Hypothesis 1*: PD and HC continue to differ even after intensive rhythmic therapies.
*Hypothesis 2*: Measures of cadence and velocity in speech correlate with those of gait in PD and HC at every measurement point.
*Hypothesis 3*: Both types of intensive rhythmic intervention (one focused on speech, the other focused on gait) improve the cadence and velocity of both speech and gait, as manifested by improvement in all measured parameters compared to the PD patients receiving no rhythmic intervention.

## Materials and Methods

### Participants

**Figure 1:**
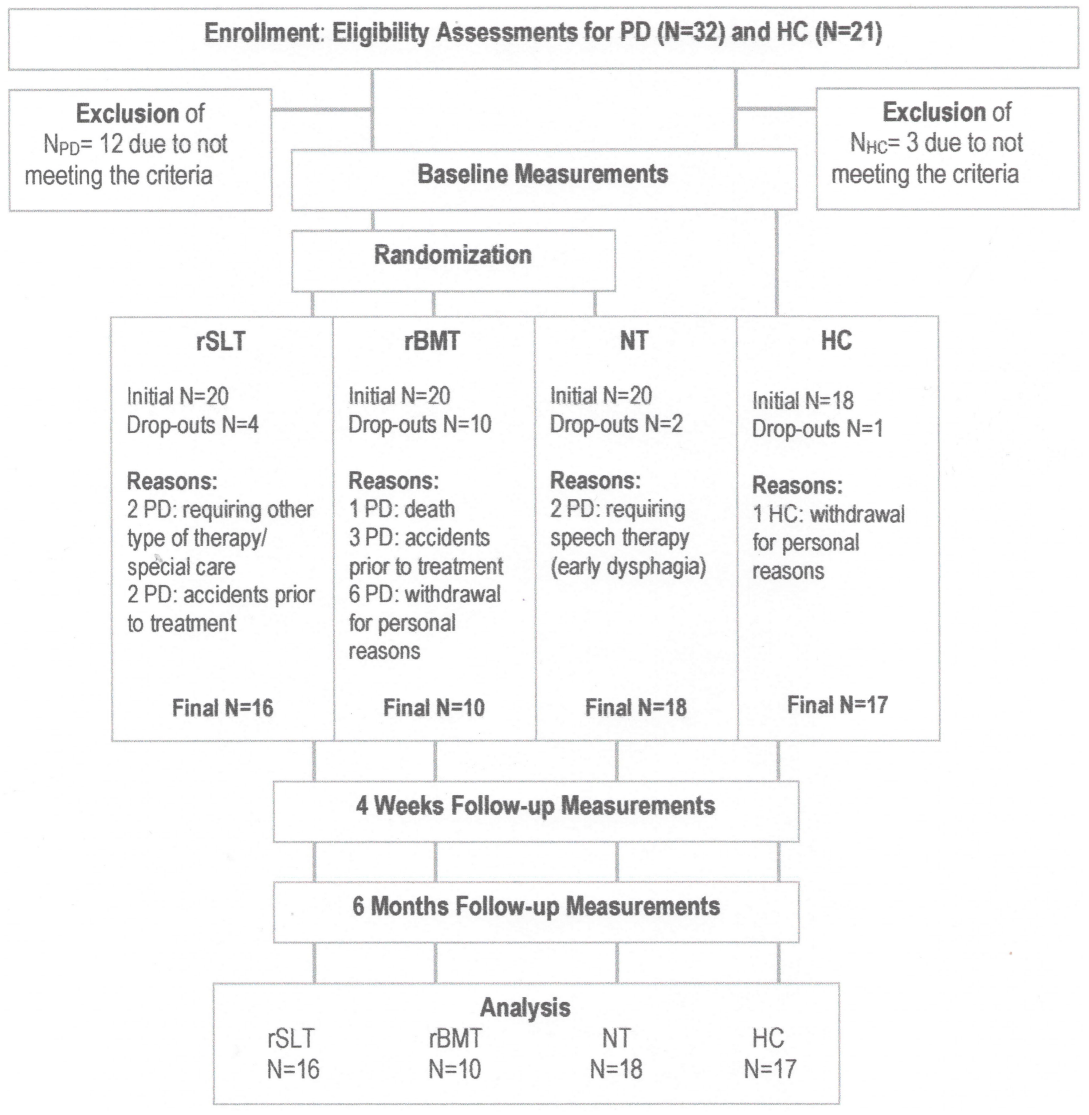
Flow chart outlining participant enrollment, treatment and assessments. Importantly, for a simplified visual overview here, all three study tranches of this crossover study have been summarized into one per study arm. The exact study outline of participant numbers per study tranche is presented in Figure 2.

From May 2015 to February 2018, 32 patients with idiopathic Parkinson's Disease (PD) were recruited from the University Hospital Basel (Switzerland) and from outside physicians, while 21 healthy controls were recruited from senior health centers separate from our hospital and from sports groups. All prospective participants were screened for eligibility by neurologists, neuropsychologists and speech-language therapists. Participants were eligible if they (a) understood and spoke German, (b) were neither demented [MoCA (98) >21] nor depressed (BDI (99) <8), and (c) were from 55 to 80 years old. The exclusion criteria were (a) secondary Parkinsonism or a concomitant disease other than PD (e.g., epilepsy, malignant tumor, severe microvascular disease, or a history of brain surgery), (b) speech-language deficits other than dysarthria, e.g., speech apraxia [*Hierarchical Word Lists* HWL (100) <85%] or aphasia of any kind [*Aachen Aphasia Test*, AAT (101) <63%], (c) having received intensive speech therapy in the past 2 years, (d) alcohol and/or drug addiction, (e) any severe mental illness (e.g., suicidality, obsessive-compulsive disorder, depression, mania, psychosis, anxiety), and (f) any other neurological or sensory problem that could interfere with the assessment. Three persons were excluded in the group of prospective healthy controls, and 12 in the group of prospective PD patient participants. Figure 1 (Flow Chart) below shows general study procedure with participant enrollment, treatments and assessments, as well as final N per group.

Importantly, as deep brain stimulation (DBS) has been reported to have mixed effects on speech deficits in PD (42, 56, 57), speech abilities of eligible PD-DBS patients were measured before and directly a week after having received DBS surgery, as well as at 3, 6, 9, and 12 months after DBS surgery. This was to monitor closely any counter effects of DBS on speech abilities. If the DBS had no effect on any measured speech parameter, PD-DBS patients were included in the study and thus, PD non-DBS and PD-DBS participants were merged into one study group.

Daily drug regimen were protocolled by patients for a week prior to baseline assessment and treatment. This was to determine ON/OFF phases and any occurring fluctuations. Assessments and therapy sessions were then only provided in ON phases. If sudden wearing-off or fluctuations would have occurred, medication would have been re-adjusted and assessments and treatments would have been re-scheduled. However, in this study levodopa dosages have been kept constant throughout the intervention periods.

Table 1 shows general demographics at baseline and at 6 months for each variable separately. Additionally, Table 1A gives *dysarthria specific speaker profiles* and Table 1B shows *motor and gait specific profiles*. Note that in Table 1A, the criteria “loudness” refers to a normal variability of loudness in speech, while “Intonation: pitch” describes the normal variability of pitch. Both characteristics were assessed during a one-on-one conversation with a normal surrounding noise level no louder than 20 dB. “Pitch” is given in Hertz (Hz) and is not reported separately for men and women. Importantly, both of these suprasegmental features of prosody are, in fact, similar in HC and PD patients, and neither appears to be affected by the intervention.

**TABLE 1:** Demographics.

	**Time**	**rSLT**	**rBMT**	**NT**	**HC**	***p-*Values across groups**
Final *N*		16	10	18	17	
*N* of PD-DBS		9	6	14		
Gender in percent (m/f)	BL	M 56% F 44%	M 50% F 50%	M 60% F 40%	M 41% F 59%	
Age in years (Mn [Range min – max]; SD)		BL	69.4 [56–79]; 7.1	69.9 [59–79]; 6.7	68.7 [56–79]; 6.7	
Education in years (Mn [Range min – max]; SD)	BL	15.1 [12–20]; 2.5	16 [12–20]; 3.2	15.1 [12–20]; 2.6	67.9 [55–75]; 5.2	*p* = 0.87
MoCA^a^ score (Mn [Range min – max]; SD)	BL	26.2 [21–29]; 2.1	27.3 [24–29]; 1.6	26.2 [21–30]; 2.2	15.5 [11–20]; 3.6	*p* = 0.86
MoCA^a^ score (Mn [Range min – max]; SD) ***p-*****Values across time**	T2	26.4 [23–30]; 1.9	27.4 [24–30]; 1.7	25.3 [20–29]; 2.5	27.9 [24–30]; 1.4	*p* = 0.42
	*p* = 0.19	*p* = 0.34	*p* = 0.07	*p* = 0.23	27.4 [25–30]; 2.9	*p* = 0.12
BDI^b^ (Mn [Range]; SD)		BL	8 [4.5–11.5]; 1.9	7.7 [4–11.5]; 2.1	7.5 [2–11.5]; 1.9	
BDI^b^ (Mn [Range]; SD) ***p-*****Values across time**	T2	7.4 [4.5–11.5]; 1.7	8.8 [7–11.5]; 1.6	8.7 [5–14]; 1.8	5.5 [0–8]; 2.2	*p* = 0.003
	*p* = 0.007	*p* = 0.084	*p* = 0.007	*p* = 0.72	5.4 [0–8]; 2.2	*p* < 0.001
PD disease duration in years (Mn [Range min – max]; SD)		BL	10.3 [4–18]; 4.2	10.4 [4–18]; 4.4	9.1 [4–18]; 3.7	
Levodopa equivalent dose (Mn [Range min – max]; SD)	BL	568 [160–1,596]; 387.4	537.9 [160–1,596]; 440.4	596.6 [160–1,596]; 431.6		
Levodopa equivalent dose (Mn [Range min – max]; SD) ***p-*****Values across time**	T2	568 [160–1,596]; 387.4	537.9 [160–1,596]; 440.4	596.6 [160–1,596]; 431.6		*p* = 0.94
		*p* = 1	*p* = 1	*p* = 1		*p* =0.94

a *Montreal cognitive assessment.*

b *Beck's depression inventory*.

**TABLE 1A:** Dysarthria specific speaker profiles.

**Variable**	**Time point**	**rSLT**	**rBMT**	**NT**	**HC**	***p-*Values across PD groups**
^a^HWL (Mn [Range min – max]; SD)	BL	98 [93.4–100]; 2.7	98.6 [93.4–100]; 2.8	99.03 [92.7–100]; 2.1	100 [0–0]; 0	*p* = 0.07
	T2	97.9 [93.6–100]; 2.8	99.5 [96.5–100]; 1.1	98.6 [90–100]; 2.8	100 [0–0]; 0	*p* = 0.06
***p-*** **Values across time**		*p* = 0.37	*p* = 0.39	*p* = 0.61	p = 1	
^b^AAT (Mn [Range min – max]; SD)	BL	93.8 [76.7–100]; 8.2	96.3 [83.3–100]; 5.3	98.1 [83.3–100]; 3.9	100 [0–0]; 0	***p*** **= 0.009**
	T2	94.8 [80–100]; 6.5	97.3 [83.3–100]; 2.1	97.1 [86.6–100]; 4.8	100 [0–0]; 0	***p*** **= 0.03**
***p-*** **Values across time**		*p* = 0.68	*p* = 0.21	*p* = 0.17	*p* = 1	
Loudness in dB (Mn [Range min – max]; SD)	BL	64.3 [45.8–79.2]; 6.9	65.3 [48.4–75.9]; 7.3	60.1 [45.4–73.2]; 9.2	65.1 [43.9–74.8]; 9.1	*p* = 0.06
	T2	65.2 [47.6–78.7]; 6.9	67.8 [49.5–74.4]; 7.3	59.6 [43.2–75.5]; 10.1	66.8 [44.8–74.7]; 3.8	***p*** **= 0.05**
***p-*** **Values across time**		*p* = 0.12	*p* = 0.05	*p* = 0.27	*p* = 0.2	
Intonation: Pitch in Hz (Mn [Range min – max]; SD)	BL	168.8 [96.6–312.5]; 47.2	178.1 [96.8–210.4]; 49.5	174.1 [96.6–318.9]; 47.5	201.8 [94.6–318.9]; 63.3	*p* = 0.29
	T2	168.9 [91.6–360.3]; 57.7	175.7 [91.6–204.2]; 50.02	172.8 [96.6–327.9]; 48.3	196.9 [97.6–327.9]; 65.02	*p* = 0.48
***p-*** **Values across time**		*p* = 0.98	*p* = 0.12	*p* = 0.25	*p* = 0.16	
^c^FDA-2 Respiration without phonation	BL	55.8 [36–72]; 13.5	54.4 [24–76]; 20.2	73.5 [52–96]; 12.9	100 [0–0]; 0	***p*** **= 0.001**
	T1	91.5 [72–100]; 10.7	50.2 [22–70]; 17.8	49.5 [36–72]; 12.9	100 [0–0]; 0	***p*** **< 0.001**
	T2	80.6 [42–96]; 15.4	43.7 [24–59]; 11.9	37.5 [24–60]; 12.9	100 [0–0]; 0	***p*** **< 0.001**
***p-*** **Values BL-T1**		***p*** **< 0.001**	*p* = 0.31	***p*** **< 0.001**		
***p-*** **values BL-T2**		***p*** **< 0.001**	***p*** **= 0.01**	***p*** **< 0.001**		
FDA-2 Respiration during phonation	BL	65.6 [54–72]; 6.7	60 [24–72]; 14.9	75.5 [48–97]; 12.1	100 [0–0]; 0	***p*** **= 0.003**
	T1	88.5 [72–100]; 11.8	75.6 [48–84]; 11.4	64.5 [36–88]; 13.4	100 [0–0]; 0	***p*** **< 0.001**
	T2	78.5 [72–96]; 14.2	49.2 [24–60]; 11.9	49.1 [12–76]; 18.5	100 [0–0]; 0	***p*** **< 0.001**
***p-*** **Values BL-T1**		***p*** **< 0.001**	***p*** **< 0.001**	***p*** **< 0.001**		
***p-*** **values BL-T2**		***p*** **= 0.001**	***p*** **< 0.001**	***p*** **< 0.001**		
FDA-2 Maximal phonation	BL	34.8 [12–68]; 17.6	35.6 [12–68]; 23.7	42.7 [12–68]; 20.3	100 [0–0]; 0	*p* = 0.48
	T1	69.9 [24–96]; 20.9	33 [12–56]; 19.3	38.1 [12–76]; 17.5	100 [0–0]; 0	***p*** **< 0.001**
	T2	57.9 [12–84]; 20.9	24.8 [12–44]; 13.03	21.3 [0–44]; 12.8	100 [0–0]; 0	***p*** **< 0.001**
***p-*** **Values BL-T1**		***p*** **< 0.001**	*p* = 0.59	***p*** **= 0.05**		
***p-*** **values BL-T2**		***p*** **< 0.001**	*p* = 0.07	***p*** **< 0.001**		
FDA-2 Spontaneous intonation	BL	53.9 [24–74]; 19.8	58.3 [48–74]; 10.2	63.8 [44–76]; 8.8	100 [0–0]; 0	*p* = 0.13
	T1	93.3 [60–100]; 13.2	66.8 [48–96]; 17.7	63.3 [12–92]; 18.4	100 [0–0]; 0	***p*** **< 0.001**
	T2	80.8 [48–98]; 21.7	62.6 [48–89]; 15.1	59.7 [44–68]; 5.9	100 [0–0]; 0	***p*** **= 0.001**
***p-*** **Values BL-T1**		***p*** **< 0.001**	*p* = 0.06	*p* = 0.9		
***p-*** **values BL-T2**		***p*** **< 0.001**	*p* = 0.25	***p*** **= 0.03**		
FDA-2 Lips	BL	58.8 [40–70]; 8.7	58.9 [44–70]; 9.4	61.9 [45–70]; 8.8	100 [0–0]; 0	*p* = 0.55
	T1	88.6 [65–100]; 11.8	67.8 [64–78]; 5.8	62.7 [45–74]; 9.6	100 [0–0]; 0	***p*** **< 0.001**
	T2	90.6 [65–100]; 12.2	58.8 [42–70]; 9.9	52.7 [35–64]; 9.6	100 [0–0]; 0	***p*** **< 0.001**
***p-*** **Values BL-T1**		***p*** **< 0.001**	***p*** **= 0.003**	***p*** **= 0.04**		
***p-*** **values BL-T2**		***p*** **< 0.001**	*p* = 0.76	***p*** **< 0.001**		
FDA-2 Tongue	BL	58.9 [40–70]; 8.7	58.9 [44–70]; 9.4	62.1 [45–70]; 8.8	100 [0–0]; 0	*p* = 0.6
	T1	88.6 [65–100]; 11.8	67.8 [64–78]; 5.7	61.9 [45–74]; 9.7	100 [0–0]; 0	***p*** **< 0.001**
	T2	89.8 [53–100]; 14.3	67.8 [64–78]; 5.8	52.6 [35–64]; 10.4	100 [0–0]; 0	***p*** **< 0.001**
***p-*** **Values BL-T1**		***p*** **< 0.001**	***p*** **= 0.003**	***p*** **= 0.04**		
***p-*** **values BL-T2**		***p*** **< 0.001**	***p*** **= 0.003**	***p*** **< 0.001**		
FDA-2 Intelligibility words	BL	87.6 [48–96]; 13.6	85.8 [48–96]; 15.8	90.1 [60–96]; 11.8	100 [0–0]; 0	*p* = 0.7
	T1	94.6 [60–100]; 10.6	82.2 [48–92]; 14.8	86.5 [56–92]; 11.6	100 [0–0]; 0	***p*** **= 0.03**
	T2	90.1 [36–100]; 18.6	71.4 [48–80]; 11.9	74.2 [44–80]; 11.2	100 [0–0]; 0	***p*** **= 0.003**
***p-*** **Values BL-T1**		***p*** **< 0.001**	***p = 0.0*** **7**	***p*** **= 0.05**		
***p-*** **values BL-T2**		*p* = 0.22	***p*** **< 0.001**	***p*** **< 0.001**		
FDA-2 Intelligibility sentences	BL	83.5 [48–96]; 16.5	83 [48–96]; 16.1	89.2 [60–96]; 11.9	100 [0–0]; 0	*p* = 0.43
	T1	91 [60–100]; 12.9	79.4 [48–92]; 15.1	85.2 [56–92]; 11.9	100 [0–0]; 0	*p* = 0.09
	T2	89 [36–100]; 21.1	68.6 [48–80]; 12.6	73.2 [44–80]; 11.9	100 [0–0]; 0	***p*** **= 0.004**
***p-*** **Values BL-T1**		***p*** **< 0.001**	***p*** **< 0.001**	*p* = 0.07		
***p-*** **values BL-T2**		*p* = 0.08	***p*** **< 0.001**	***p*** **< 0.001**		
FDA-2 Intelligibility spontaneous speech	BL	79.1 [48–96]; 19.9	79.6 [48–96]; 18.8	88.2 [60–96]; 12.3	100 [0–0]; 0	*p* = 0.12
	T1	87.1 [60–100]; 16.4	76 [48–92]; 18.1	84.8 [56–92]; 12.3	100 [0–0]; 0	*p* = 0.19
	T2	85.9 [36–100]; 24.1	65.2 [44–80]; 16.3	72.8 [44–80]; 12.3	100 [0–0]; 0	***p*** **= 0.02**
***p-*** **Values BL-T1**		***p*** **< 0.001**	***p*** **< 0.001**	*p* = 0.12		
***p-*** **values BL-T2**		***p*** **= 0.05**	***p*** **< 0.001**	***p*** **< 0.001**		

*BL, baseline; T1, 4 weeks follow-up; T2, 6 months follow-up;*

a *HWL, Hierarchical Wordlist (German speech apraxia test);*

b *AAT, Aachen Aphasia Test (German standard aphasia test).*

c *FDA—Frenchay Dysarthria Scale (German Version); Standard 8-point-scale scoring sheet was transferred into percent according to formula: 100%/8 = 12.5%, and subsequently: 12.5%/10 = 1.25%.*

*FDA is a perceptually based assessment where the SLT rates the patient's abilities when performing specific tasks.*

*>87% = no impairment.*

*<87% = inconsistently occurring mild impairment.*

*<65.5% = mild impairment.*

*<50% = moderate impairment.*

*<37.5% = inconsistently occurring severe impairment.*

*<25% = consistently occurring severe impairment.*

*<12.5% = impairment disabling patient to complete task.*

Bold values are significant values as in : ^***^p < 0.001; ^**^p < 0.01; and ^*^p < 0.05.

**TABLE 1B:** Motor and gait specific profiles.

**UPDRS subscales**		**rSLT**	**rBMT**	**NT**	**HC**	***p-*values across PD groups**
I	BL	1.3 [0–4]; 1.3	1.2 [0–3]; 1.03	1.2 [0–5]; 1.2		*p* = 0.93
II	BL	11.6 [4–20]; 4.2	12.8 [6–20]; 4.7	12.7 [5–22]; 4.4		*p* = 0.73
III	BL	23.8 [6–48]; 12.1	19 [0–46]; 13.7	23.8 [7–46]; 10.8		*p* = 0.54
IV	BL	4.1 [0–13]; 3.5	4.3 [2–13]; 3.5	4.2 [0–13]; 3		*p* = 0.98
UPDRS total (Mn [Range min – max]; SD)	BL	40.8 [10–83]; 18.7	37.3 [12–81]; 20.6	41.8 [12–81]; 17.3		*p* = 0.82
	T2	37.3 [7–69]; 16.3	41.7 [19–77]; 19.1	39.6 [16–71]; 16.9		*p* = 0.81
***p-*** **values across time**		*p* = 0.3	*p* = 0.32	*p* = 0.31		
^a^POMA/Tinetti	BL	18 [6–28]; 5.3	17.7 [3–26]; 6.4	18.6 [13–28]; 3.9		*p* = 0.56
	T1	18.7 [9–28]; 5.8	18.3 [8–22]; 5.4	18.8 [10–28]; 4.2		*p* = 0.7
	T2	17.8 [6–28]; 6.4	17.9 [11–24]; 4.3	17.2 [11–26]; 4.3		*p* = 0.86
***p-*** **values across time**		*p* = 0.64	*p* = 0.62	*p* = 0.65		
One-Leg Stand Balance Test (Mn [Range min – max]; SD) in sec	BL	15.8 [0–121]; 29.4	22.1 [2–105]; 36.3	15.7 [0–68];15.8	63.4 [3–270]; 68.1	***p*** **< 0.001**
	T1	23.9 [0–105]; 33.6	31.8 [2–114]; 37.6	19.8 [3–100]; 23.3	63.6 [11–270]; 59.2	***p*** **< 0.001**
	T2	19.3 [0–95]; 26.3	12 [0–28]; 10.8	13.3 [3–45]; 12.9	81.8 [15–270]; 82.3	***p*** **< 0.001**
***p-*** **values across time**		*p* = 0.1	***p*** **< 0.05**	*p* = 0.2	***p*** **< 0.01**	
Tandem Stand Balance Test (Mn [Range min – max]; SD) in sec	BL	30.3 [0–126]; 39.7	40.3 [0–142]; 50.1	23.8 [0–162]; 36.8	78.2 [3–205]; 60.9	***p*** **< 0.001**
	T1	37.7 [0–142]; 50.4	44.8 [2–120]; 44.8	22.5 [2–178]; 40.2	120.1 [5–730]; 172.7	***p*** **< 0.001**
	T2	33.6 [0–162]; 43.1	37.8 [0–127]; 46.4	18.3 [0–51]; 16.6	176 [3–590]; 144.3	***p*** **< 0.001**
***p-*** **values across time**		*p* = 0.3	*p* = 0.37	*p* = 0.22	***p*** **< 0.01**	
^b^FES-I (Mn [Range min – max]; SD)	BL	20.1 [16–59]; 10.8	22.5 [16–31]; 9.2	17.6 [16–40]; 5.7	16 [0–0]; 0	*p* = 0.35
	T1	20.3 [16–64]; 12	18.2 [16–26]; 3.9	20.7 [16–41]; 9.6	16 [0–0]; 0	*p* = 0.8
	T2	22.8 [16–64]; 14.2	22.6 [16–59]; 13.8	19.8 [16–49]; 8.4	16 [0–0]; 0	*p* = 0.73
***p-*** **values BL-T1**		*p* = 0.59	*p* = 0.08	*p* = 0.13	*p* = *1*	
***p-*** **values BL-T2**		*p* = 0.26	*p = 0.9*8	*p* = 0.07		
^c^FGA-I *Task 1*	BL	1.84 [0–3]; 0.83	1.63 [0–2]; 0.67	1.9 [0–3]; 0.8		*p* = 0.7
	T1	2.1 [0–3]; 0.87	2.09 [0–3]; 0.9	1.6 [0–3]; 0.8		***p*** **= 0.05**
	T2	1.82 [0–3]; 0.83	1.73 [0–2]; 0.7	1.7 [0–3]; 0.9		*p* = 0.9
***p-*** **values BL-T1**		***p*** **= 0.02**	***p*** **= 0.01**	*p* = 1		
***p-*** **values BL-T2**		*p* = 0.9	*p* = 0.8	*p* = 0.6		
^c^FGA-I *Task 2*	BL	1.2 [0–2]; 0.85	1.3 [0–2]; 0.7	1.5 [0–3]; 1.01		*p* = 0.8
	T1	1.7 [0–3]; 1.06	1.5 [0–3]; 0.9	1.5 [0–3]; 1.01		*p* = 0.8
	T2	1.2 [0–2]; 0.83	1.4 [0–2]; 0.7	1.2 [0–3]; 0.9		*p* = 0.7
***p-*** **values BL-T1**		***p*** **= 0.002**	***p*** **= 0.05**	*p* = 1		
***p-*** **values BL-T2**		*p* = 1	*p* = 0.07	*p* = 0.06		
^c^FGA-I *Task 3*	BL	1.1 [0–2]; 0.73	1.4 [0–2]; 0.7	1.2 [0–2]; 0.8		*p* = 0.7
	T1	1.1 [0–2]; 0.73	1.4 [0–2]; 0.7	1.2 [0–2]; 0.8		*p* = 0.7
	T2	1.1 [0–2]; 0.73	1.4 [0–2]; 0.7	0.95 [0–2]; 0.8		*p* = 0.06
***p-*** **values BL-T1**		*p* = 1	*p* = 1	*p* = 1		
***p-*** **values BL-T2**		*p* = 1	*p* = 1	*p* = 0.08		
^c^FGA-I *Task 4*	BL	1.2 [0–2]; 0.85	1.3 [0–2]; 0.6	1.5 [0–3]; 1.01		*p* = 0.9
	T1	1.2 [0–2]; 0.85	1.4 [0–2]; 0.7	1.5 [0–3]; 1.01		*p* = 0.9
	T2	1.2 [0–2]; 0.85	1.4 [0–2]; 0.7	1.1 [0–2]; 0.97		*p* = 0.9
***p-*** **values BL-T1**		*p* = 1	*p* = 0.84	*p* = 1		
***p-*** **values BL-T2**		*p* = 1	*p* = 1	*p* = 0.08		
^c^FGA-I Task 5	BL	1.6 [0–3]; 1.01	1.4 [0–3]; 0.8	1.4 [0–3]; 0.95		*p* = 0.8
	T1	1.6 [0–3]; 1.07	1.4 [0–3]; 0.8	1.4 [0–3]; 0.95		*p* = 0.8
	T2	1.6 [0–3]; 1.07	1.4 [0–3]; 0.8	1.01 [0–2]; 0.8		*p* = 0.5
***p-*** **values BL-T1**		*p* = 1	*p* = 1	*p* = 1		
***p-*** **values BL-T2**		*p* = 1	*p* = 1	***p*** **= 0.04**		
^c^FGA-I *Task 6*	BL	1.6 [0–3]; 0.95	1.5 [0–2]; 0.7	1.6 [0–3]; 1.1		*p* = 0.9
	T1	1.9 [0–3]; 1	1.9 [0–3]; 1.04	1.6 [0–3]; 1.1		*p* = 0.9
	T2	1.6 [0–3]; 0.95	1.5 [0–2]; 0.68	1.4 [0–3]; 1.04		*p* = 0.8
***p-*** **values BL-T1**		*p* = 0.07	*p* = 0.08	*p* = 1		
***p-*** **values BL-T2**		*p* = 1	*p* = 1	*p* = 0.08		
^c^FGA-I Task 7	BL	1.6 [0–3]; 1.07	1.4 [0–3]; 0.8	1.4 [0–3]; 0.96		*p* = 0.9
	T1	1.8 [0–3]; 1.2	1.4 [0–3]; 0.8	1.3 [0–3]; 0.8		*p* = 0.9
	T2	1.6 [0–3]; 1.2	1.4 [0–3]; 0.8	1.1 [0–3]; 0.95		*p* = 0.7
***p-*** **values BL-T1**		*p* = 0.08	*p* = 1	*p* = 1		
***p-*** **values BL-T2**		*p* = 1	*p* = 1	*p* = 0.3		
^c^FGA-I *Task 8*	BL	1.1 [0–2]; 0.73	1.3 [0–2]; 0.65	1.3 [0–2]; 0.84		*p* = 0.9
	T1	1.1 [0–2]; 0.73	1.5 [0–3]; 0.9	1.3 [0–2]; 0.83		*p* = 0.7
	T2	1.1 [0–2]; 0.73	1.3 [0–2]; 0.65	0.95 [0–2]; 0.78		*p* = 0.5
***p-*** **values BL-T1**		*p* = 1	*p* = 0.4	*p* = 1		
***p-*** **values BL-T2**		*p* = 1	*p* = 1	*p* = 0.9		
^c^FGA-I Task 9	BL	1.6 [0–3]; 1.1	1.4 [0–3]; 0.8	1.4 [0–3]; 0.96		*p* = 0.9
	T1	1.6 [0–3]; 1.1	1.5 [0–3]; 0.93	1.4 [0–3]; 0.96		*p* = 0.8
	T2	1.6 [0–3]; 1.1	1.4 [0–3]; 0.8	1.1 [0–3]; 0.1		*p* = 0.8
***p-*** **values BL-T1**		*p* = 1	*p* = 0.76	*p* = 1		
***p-*** **values BL-T2**		*p* = 1	*p* = 1	*p* = 0.07		
^c^FGA-I Task 10	BL	1.2 [0–2]; 0.73	1.4 [0–2]; 0.7	1.5 [0–3]; 1.01		*p* = 0.8
	T1	1.2 [0–2]; 0.85	1.4 [0–2]; 0.7	1.5 [0–3]; 1.01		*p* = 0.8
	T2	1.2 [0–2]; 0.84	1.4 [0–2]; 0.6	1.5 [0–3]; 1.01		*p* = 0.8
***p-*** **values BL-T1**		*p* = 1	*p* = 1	*p* = 1		
***p-*** **values BL-T2**		*p* = 1	*p* = 1	*p* = 1		

*BL, baseline; T1, 4 weeks follow-up; T2, 6 months follow-up.*

a *POMA–Parkinson orientated Mobility Assessment (Falls risk assessment awards points for the patient's postural stability when sitting, standing up, walking, and performing a 360° turn). Fall-risk scores range from 0 to 28: 25–28 (low risk), 19–24 (medium risk), <19 (high risk).*

b *FES-I**—**Falls Efficacy Scale—International (standard questionnaire to measure fear of falling in elderly). Scoring ranges from 16 to 64: 16–19 (low concern), 20–27 (medium concern), 28–64 (high concern).*

c *FGA-I—Functional Gait Assessment- International (standard measurement to evaluate postural stability during walking and performing multiple motor tasks while walking. Marked walkway 6 m long and 30.48 cm wide). Scoring ranges from 0 to 3:*

*0—severe impairment, cannot perform without assistance, severe gait deviations, or imbalance.*

*1—moderate impairment.*

*2—mild impairment.*

*3—no gait or balance impairment, task completion within given time and marked walking area.*

*Bold values are significant value*.

**Brief description of dysrhythmic deficits in PD patients:**

Festinations and freezing occurring in both, speech and gait.*Gait-specific description*: Shuffled and accelerated steps, short stride length, asymmetrical or missing arm-leg swing.*Speech-specific description*: Asymmetrical breathing control and/or breathing movements are asynchronous, speaking pauses are out of place and distorting longer speaking sequences resulting in rushed speaking, unintelligible articulation, missing accentuations, monotone speech rate. Importantly, speaking loudness and intonation is within “healthy” ranges.

### Determining Sample Size

Group sizes were determined on the basis of a priori analysis using G^*^power analysis© (computer software program, Version 2014). Our input parameters for the calculation of *required sample size* were: moderate to strong effect size (Cohen's *d* ≥ 0.45); high statistical power (1-β error of probability = 0.80), a high level of significance (α ≤ 0.005), with four groups (i.e., rSLT, rBMT, NT, and HC) and for at least 3 measurements (BL and at both follow-ups being T1-4 weeks and T2–6 months). These input parameters are similar to other motor (102, 103) or speech (38) intervention studies. Our analysis resulted in a total sample size of 40 participants, being 10 participants per study group.

As we expected a drop-out rate of possibly up to 50% per group, we planned a group size of at least 20 participants per group. Figure 1 below contains a flow chart of patient enrollment, rationale of drop-outs per study group, and treatment and assessment procedures.

Similarly, replicating a G^*^power analysis on the basis of given core values (i.e., total sample size, number of measurements and number of groups, employed statistical tests) deriving from several motor intervention studies (102, 103) and a recent intensive speech intervention study (38) involving 10-20 participants per study arm, the statistical analysis revealed equally moderate effect sizes (Cohen's *d* ≥ 0.45).

### Study Design

**Figure 2:**
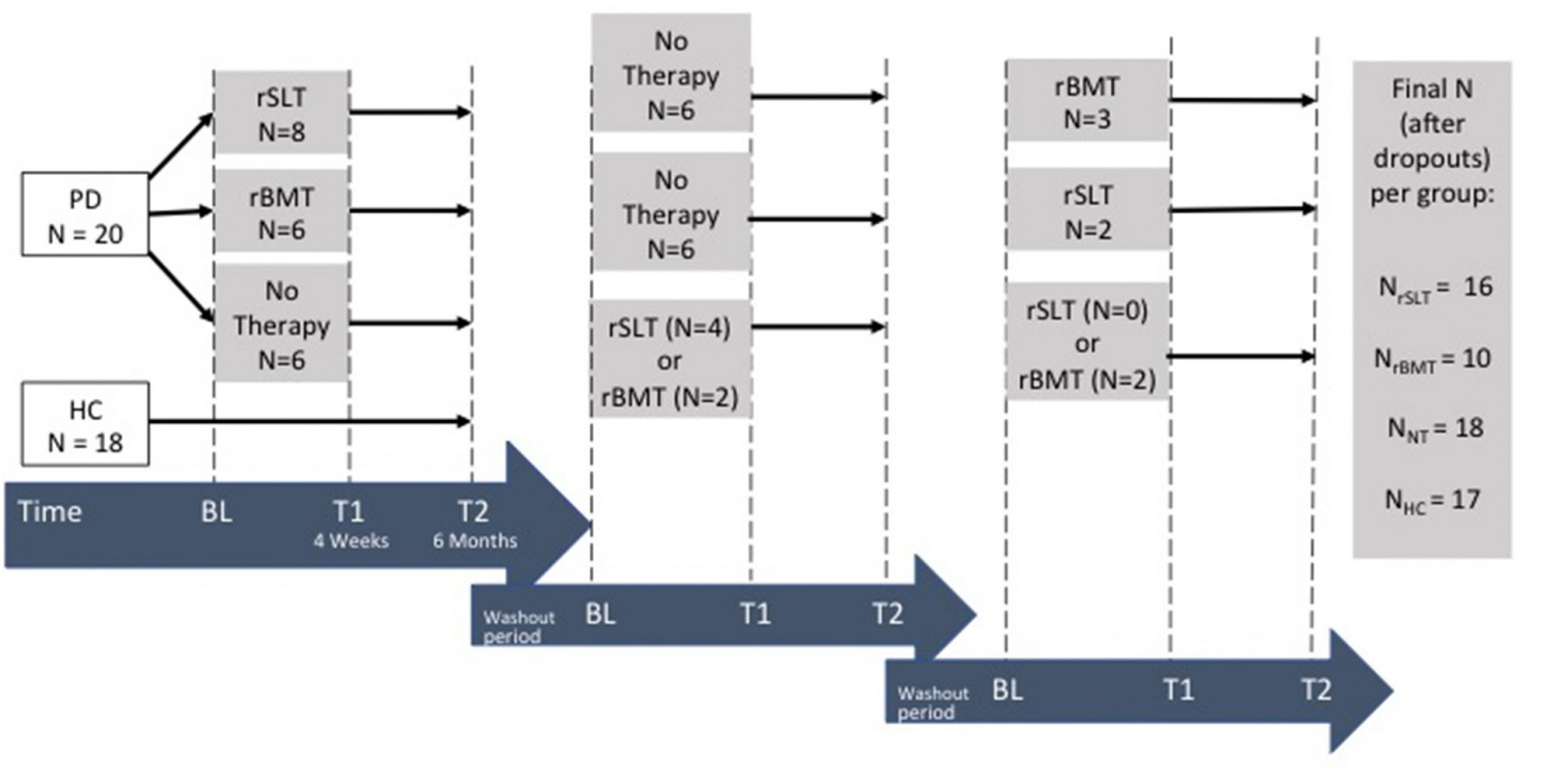
Study design.

The crossover design included three parallel, controlled, and single-blinded study arms (Figure 2). In this crossover study, PD patients were able to participate in all three different study conditions (i.e., rSLT, rBMT, or no therapy) upon completion of the 6-months study period, and if they have not undergone the same treatment before. Although, the participation in all three PD study conditions (i.e., rSLT, rBMT, and NT) was anticipated, the completion of all three study conditions was not compulsory. Participants could drop-out or decline from participation at any time point. Figure 2 holds the exact participant numbers per study tranche and PD group. Importantly, the HCs received no intervention and as thus, participated only once in the study.

After baseline evaluations, the PD patients were randomly allotted to the groups (i.e., rSLT, rBMT, and NT), with group matching for sex, age, and educational level. There were three intervention tranches in total, with changing of the therapists and study personnel between tranches. As in cross-over studies results may be affected by carry-over effects, a wash-out period of 6 months (being the same length as the treatment period) was planned after every treatment period (104–106).

Further, in order to preserve examiner blinding (a) therapists providing interventions (rSLT, rBMT) did not evaluate the patients that they themselves treated, (b) assessments (BL, T1-4 Weeks, and T2-6 Months) were done by an independent study assessment personnel, which did not know to what group patients were allocated to, and (c) both interventions were provided by speech-language therapists. Reasons for the latter were to strengthen examiner-blinding in that, undoubtedly anyone, who had been trained previously how to provide the rBM-Treatment and fill out performance protocol forms, could provide this form of treatment. However, if both treatments (rSLT and rBMT) were provided by speech-language therapists, then the independent study assessment personnel could not trace back what treatment has been provided by seeing which therapist had signed up the patient to be assessed at T1 (4 Weeks) or T2 (6 Months).

### Standard Protocol Approvals, Registrations, and Patient Consents

The study was registered with Clinicaltrials.gov (NCT 029 358 42). Ethical approval was obtained from the “Ethikkommission Nordwest- und Zentralschweiz” (EKNZ), No. 2016-01 428. All participants were fully informed of the nature and scope of the study, and its possible effects and risks. Before participation, all participants gave their written consent.

### Interventions

Both interventions, rSLT and rBMT, were administered by professionally trained speech-language therapists. Therapists were compliant with the “clinical trial unit” (CTU) requirements of the University Hospital and were trained according to the “ambulatory study center” (ASZ) standards of clinical research. Detailed outline and treatment protocols are given in Table 2. These two interventions are similar at the level of intensity and frequency (i.e., being provided 3x per week in 45 min-long training sessions, for 4 weeks in total) both including high-effort exercises targeting motor and sensory retraining. The difference between these two treatments lies in the specificity of the method: a rhythmic balance-mobility specific vs. a rhythmic speech-language specific approach. In more detail, the rBMT targets to improve balance and mobility, and is delivered via a computerized rhythmic step-training program. In contrast, the rSLT targets to improve speech rhythm and intelligibility via rhythmic accentuations focusing on motor-articulation, breathing control and speech rhythm.

**TABLE 2:** Descriptions, outline, and protocols of treatments.

	**rSLT**	**rBMT**
Target of therapy	Re-establishing naturally rhythmic speech via self-perception of respiration, speed of speaking, accentuations, and pauses	Balance-Mobility, reducing falls risk, and fear of falls
Origin of method	“Accent Method” (107)	REDANCE^®^ (by Dinevski and Schulte) (108)
Setting		
	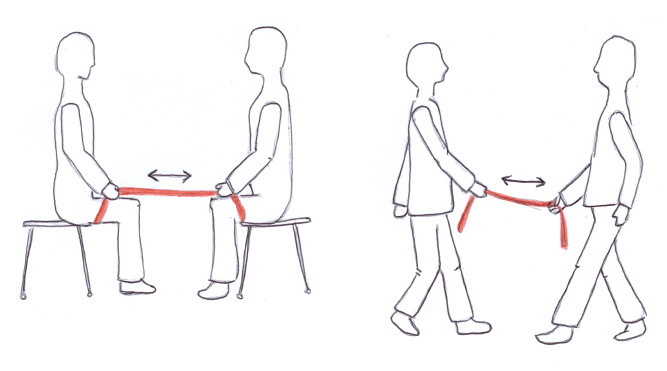 • In a sitting or standing position • Quiet room at clinic • One-to-one situation • Patient directly opposite of therapist	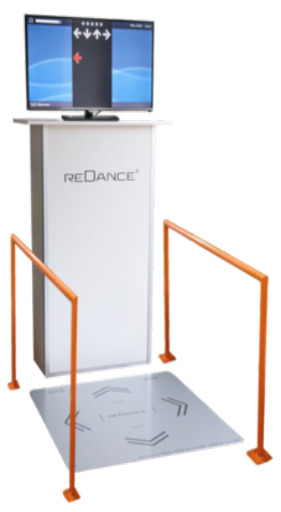 • Quiet room at clinic • Patient opposite of screen, standing on dance platform • Personnel only present to set up training devices, fill out dance performance protocol forms, and assist in any case of emergency
Supporting material(s)	• Rhythmic swinging (Impulse) via red Thera-band^®^ (approx. 150 cm long) • Method allows direct and encouraging feedback • Patient imitates directly after therapist exemplifies accentuated phonation exercise • Daily exercise routine (patient-individual); Motto: “*use it or lose it*”	• Music played via TV • Arrows on screen indicating time and type of movements • 82.5 cm wide and long, and 2.2. cm high dance platform with a fixed and immobile hip-high handrail to the left and right side of the platform
Type of feedback	• Verbal direct corrective feedback from the therapist	• 5 stars in a row at the top of the screen • After every step stars light up in accordance to accuracy of steps (i.e. accuracy of steps were measured regarding exact timing and correct position of the step).
Intensity and Frequency	45 minute sessions, 3x per week for 4 weeks in total	
1 week	• Self-perception of respiration and posture • Modulation of behavior: re-establishing symmetric and synchronized breathing movements and abdominal breathing complement (control of diaphragm) • Accentuations in tempi *Largo* on phonation of/w/	• 10–15 dances on Level 1 • Max. Length of 2–3 min per song • Involving 70–140 steps per song
2 week	• Respiration exercises • Accentuations in tempi *Andante* • String of phonations: /w/, /wo/, /wu/ and /wa/, then on phonation of mono-syllabic word strings (e.g., /where?/, /when?/, /why?/, /wall/, will/, /wand/, /wind/,/wound/) • Only words with soft endings	• If 3 dances on Level 1 exceeded 70% of performance, than moving on to Level 2 • 10–15 dances (Level 1 or Level 2) • Max. Length of 2–3 min per song • Involving 90–180 steps (per song) on Level 2
3 week	• Respiration Exercises • Accentuations in tempo *Andante* on phonations of 1–2 syllabic word strings (/whisper/, /worry/, /willing/, etc.) • 3 Variations in intonation and prosody	• 10–15 dances on Level 2 or if completing 3 dances with > 70% on Level 2, then moving on to Level 3 • Max. Length of 2–3 min per song • Involving 90–180 steps (per song) on Level 2 or 190–230 steps per song on Level 3
4 week	• Accentuations in tempi *Allegro* on 5–8 word sentences • Variations in intonation and prosody, pauses, and speed • Transfer to natural speaking	• 10–15 dances on Level 3 • Max. Length of 2–3 min per song • Involving 190–230 steps per song

*Rationale: Why was the single-dancer, multi-directional computer-based step-training chosen for the rBMT?* As a fact, every participant with PD comes with an individual symptom severity and mobility-balance ability. Therefore, to maximize the possible benefit for every participant, we provided individual one-on-one sessions instead of group sessions. However, costs of hiring a professional dance teacher for individual lessons could not be covered by the limited budget. Additionally, the computer-dance program gave for every individual participant a numerical performance protocol and followed a stringent training regimen (see Table 2*, weekly training*), which both would not have been the case in traditional dance classes. In sum, the computer-based step-training enabled comparisons easily, did not overstress the budget and still was provided on an individual basis. To-date, the here chosen rBMT method has shown beneficial effects in healthy elderly in terms of improving balance and mobility and reducing falls (108). Yet to this day, how the rBMT may alleviate symptoms in speech and gait in PD remains uninvestigated.

*Rationale*: *Why was the “Accent method”* *(**107**)* *chosen for the rSLT?* Speech abilities needed for everyday communication are very different from singing *per se* (94, 96), and the effect of singing on speech abilities, articulation, and breathing was not univocal (94, 96). In contrast, rhythmic speech cueing (RSC) controls speech rate via metronome beats or patterned speech. Yet, RSC has been reported to improve intelligibility most in severe Parkinsonian speech, while holding only limited benefits for mild to moderate Parkinsonian speech (97). As this studied population presents mild to moderate dysarthria with symptoms of functional dysphonia (e.g., hoarseness, huskiness, and roughness of the voice) combined with non-physiological muscle tone (e.g., rigid-hypokinetic, bradykinetic or ataxic dysarthria) the “Accent method” has been chosen. The “Accent method” (107) re-rhythmizes the patient via awareness and control of the breathing and phonation movements, and has shown beneficial effects in patients with functional dysphonia (109, 110), and in patients with non-organic voice disorders (111) and in patients with severe muscle tension dysphonia (112, 113). To-date, whether the accent method may even address a specific type of dysarthria such as in dysrhythmic PD patients remains hypothetical.

### Evaluations

Evaluations were performed at baseline (BL), directly after completion of intensive interventions at 4 weeks (T1) and again at 6 months (T2) (Figure 2). All evaluations consisted of a speech-language test battery, a neuropsychological/ -psychiatric test battery, and a variety of motor evaluations, which—except for verbal learning and word retrieval tasks were kept the same for every measurement. In this paper, we report speech metric measurements, as well as mobility and balance evaluations.

All evaluations were administered according to a predefined standard operation procedure (SOP) protocol, in a quiet clinical examining room of the University Hospital. There the participant sat in a comfortable chair at a table with the examiner sitting opposite. All linguistic evaluations were administered by professionally trained speech-language therapists (SLT), who did not know which therapy program the patient received.

*Speech assessments* included speech cadence (rhythm), speech velocity (speaking rate) and respiration during speaking. Speech respiration was added because speech cadence and velocity depend on the speaker's control of breathing. Speech rhythm was measured with the speech index of rhythmicity (SPIR) referring to the total amount of speech inter-pauses per minute. Speech velocity was measured by counting the number of accentuated syllables spoken within 1 s (SylSec), whilst speech respiration is described by the count of syllables spoken within one respiration phase (SylIns). For this purpose, the patient was asked to read a short text (“the north wind and the sun,” see Appendix 1) out loud and was recorded with an Olympus WS-850 recorder 30 centimeters distant from the mouth (114, 115). Speech recordings were sampled at 48 kHz with 16-bit resolution. For additional speech analysis, the mean and range of loudness in decibels (dB), the variability of pitch in hertz (Hz) and the duration of phonation of accentuated syllables in seconds were measured with the aid of PRAAT^®^ software. Results concerning loudness and pitch are presented in Table 1, as these variables are part of the sample description. From among all of the speech recordings, 20 were randomly chosen to be re-analyzed by a second independent SLT. Spearman's correlation analysis between the two sets of independent ratings showed good agreement (*r* = 0.859, *p* < 0.001).

*Motor assessments* involved the stand-up-and-go-test (TUG) and the first part of the functional gait assessment (116) (FGA).

For the TUG, participants were asked to stand up from a sitting position (after hearing the starting sound), walk a distance of three meters, turn around, walk back the same way and sit back down again. Participants were encouraged to do this exercise as fast as possible, and the time was recorded with a stopwatch.

For the FGA (116), the participants had to walk 6 m at their normal individual pace. The path was marked with a measurement band on the floor all along the 6 m walkway, and at a fixed width of 30.48 centimeters at both sides. Spatio-temporal parameters measured included *velocity* (i.e., walking speed given in meters per second [m/s]), *cadence* (i.e., number of steps per second), and step length (given in millimeters [mm]). A step is defined as an individual contact of one foot with the ground, while step length is the linear distance between the contacts of the heels of one foot and the opposite limb. For the kinematic analysis, the participant's knee and feet were marked with highlighting stickers, and walking sequences were videotaped. Video recordings were at a sampling rate of 100 Hz.

### Statistical Procedures

In order to verify that no other concomitant speech or cognitive disorder arose over the study period, all variables used to describe the sample characteristics have been measured at baseline and at 6 months and several intergroup *t-tests* have been conducted per study group (see Tables 1A,B).

Furthermore, to control for group bias, several *univariate ANOVAs* with *post-hoc* Bonferroni corrections were performed across groups (i.e., rSLT, rBMT, NT, HC) for the following variables: age, education, other speech-language deficits [i.e., speech apraxia (100), aphasia (101), deficits in loudness or pitch], cognitive abilities [MoCA (98)], depression [BDI (99)], motor abilities [i.e., UPDRS (117)], risk of falling [i.e., POMA (118)], balance tests (i.e., one-leg and tandem standing test), medication dosage and PD-disease duration. In the case of the PD groups, if these showed no significant intergroup differences, they were merged into one group and compared against HCs with the aid of *independent t-tests*.

Relationships between speech and gait variables were computed with *Spearman's correlation*s (significance level *p* < 0.05). Measures of velocity (i.e., syllables per second [SylSec] and meters per second [m/s]) and cadence (i.e., amount of inter-speech pauses per minute [SPIR] and steps per minute [steps/min]) were correlated with each other. The level of statistical significance was set at *p* < 0.05.

Changes in performance were analyzed with the reliable change score (RC). According to Jacobson and Truax (119), scores are compared on a participant-individual level [e.g., BL with T1-4 weeks Follow-up (FU), and BL with T2-6 months Follow-up (FU)]. Positive scores indicate an improvement in performance, and negative scores indicate a worsening of performance. The RC shows whether a difference in performance is statistically significant on the basis of the reliability of the measurement. Further, the reliable change index (RCI) provides the cut-off at which a performance can be seen as statistically significant. The RCI is based on the division of RC scores from one participant by the *standard error of measurement of difference*. The *standard error of measurement of difference* derives from the *generic variance* and the *retest reliability* of the employed assessment. Thus, RCI values are equivalent to standardized *z-*scores, and build the cut-off to describe a significant change in performance.

For group comparisons (i.e., rSLT, rBMT, NT, and HC), *several repeated measures ANOVA* with RC scores from speech and balance-mobility as the within-group factors and with group as the in-between factor were conducted. Subsequently, *dependent* and *independent samples t-tests* were computed, if interactions flagged as significant. All statistical analyses were conducted with SPSS. All results are presented with effect sizes.

### Data Availability Statement

For the purpose of data transparency, the data used in this study (including study protocol and case report forms) will be made available on the public repository dataverse.harvard.edu. The Department of Clinical Research (DKF) of the University of Basel will act as an independent Data Access Committee (DAC) and store the data at time of publication. Sensitive data cannot be accessed via *dataverse* and is to be requested via the contact form of the DKF at https://dkf.unibas.ch/en/about-us/contact/.

## Results

### Intergroup Differences During Study Period

*One sample t-tests* computed for every variable across time (i.e., BL and 6 months Follow-up) of the sample description (Table 1) and for every individual group (i.e., rSLT, rBMT, NT, and HC), suggested no significant intergroup differences. Therefore, any concomitant diseases or other cognitive disorders possibly arising during the study period can be ruled out.

### Differences Between Groups at Baseline

No significant differences were found between PD groups (rSLT, rBMT, and NT) across variables (*p* > 0.43). HCs outperformed PD patients across variables (*p* < 0.01). Results are given in Table 3 below.

**TABLE 3:** Group bias at baseline given per variable.

**Variables**	**Groups**	**Mn [Range]; SD**	***p*-values (across PD groups)**	***p*-values (PD vs. HC)**
SPIR (speech rhythm)	rSLT	18.9 [16–22]; 1.6	*p* = 0.32	*p* < 0.001
	rBMT	18.6 [16–22]; 2.5		
	NT	17.8 [16–22]; 1.9		
	HC	10.1 [8–12]; 1.6		
SylSec (speech velocity)	rSLT	5.3 [4.2–6.9]; 0.6	*p* = 0.67	*p* < 0.001
	rBMT	5.3 [4.1–6.3]; 0.6		
	NT	5.1 [4–5.9]; 0.4		
	HC	2.3 [1.2–3.5]; 0.6		
SylINS	rSLT	8.4 [4–19]; 3.6	*p* = 0.94	*p* < 0.001
	rBMT	9 [4–19]; 3.9		
	NT	8.6 [4–20]; 4.1		
	HC	21.6 [14–36]; 6		
TUG	rSLT	18.3 [9–43]; 10.2	*p* = 0.85	*p* = 0.001
	rBMT	16.3 [7–60]; 16.1		
	NT	16.7 [9–25]; 4.7		
	HC	6.6 [4–11]; 2.4		
Walking cadence (steps/sec)	rSLT	1.8 [1–2.9]; 0.8	*p* = 0.8	*p* < 0.001
	rBMT	1.7 [1.11–2.4]; 0.8		
	NT	1.9 [1.07–2.5]; 0.7		
	HC	1.69 [1.5–2.11]; 0.18		
Walking velocity (m/s)	rSLT	0.74 [0.4–1.05]; 0.2.4	*p* = 0.88	*p* < 0.001
	rBMT	0.81 [0.5–1.05]; 2.7		
	NT	0.8 [0.5–1.05]; 3.1		
	HC	1.3 [1.1–1.4]; 0.13		
Step length (mm)	rSLT	487 [400–602]; 52.1	*p* = 0.67	*p* < 0.001
	rBMT	485 [448–619]; 87.8		
	NT	492 [400–608]; 75.4		
	HC	580 [532–625]; 50.9		

### Correlations Between Speech and Gait

Across groups and time measurement points, measures of cadence (speech [SPIR] and gait [steps/min]) were significantly positively correlated (*p* < 0.01), as were measures of velocity (speech [SylSec] and gait [m/s]) (*p* < 0.01). Table 4 shows all correlations.

**TABLE 4:** Correlations between *speech* and *gait* across groups and time.

		**rSLT**	**rBMT**	**NT**	**HC**
BL	Cadence	*r* = 0.99; *p* = 0.002	*r* = 0.95; *p* < 0.001	*r* = 0.84; *p* = 0.002	*r* = 0.93; *p* < 0.001
	Velocity	*r* = 0.86; *p* < 0.001	*r* = 0.73; *p* = 0.017	*r* = 0.66; *p* = 0.003	*r* = 0.94; *p* < 0.001
T1 (4 weeks)	Cadence	*r* = 0.84; *p* = 0.05	*r* = 0.86; *p* = 0.002	*r* = 0.85; *p* = 0.003	*r* = 0.91; *p* < 0.001
	Velocity	*r* = 0.75; *p* < 0.001	*r* = 0.74; *p* = 0.014	*r* = 0.54; *p* = 0.02	*r* = 0.93; *p* < 0.001
T2 (6 months)	Cadence	*r* = 0.95; *p* = 0.02	*r* = 0.83; *p* = 0.003	*r* = 0.72; *p* = 0.02	*r* = 0.9; *p* < 0.001
	Velocity	*r* = 0.92; *p* < 0.001	*r* = 0.95; *p* < 0.001	*r* = 0.88; *p* < 0.001	*r* = 0.93; *p* < 0.001

*Cadence in gait is given as steps per second [steps/sec]; velocity in gait is given as meters per second [m/s]; cadence in speech (speech rhythm) is measured by total amount of speech inter-pauses per minute [SPIR]; speech velocity presents syllables spoken within one second [SylSec]*.

### Comparison of Group Performances Across Time (RC)

**Figure 3:**
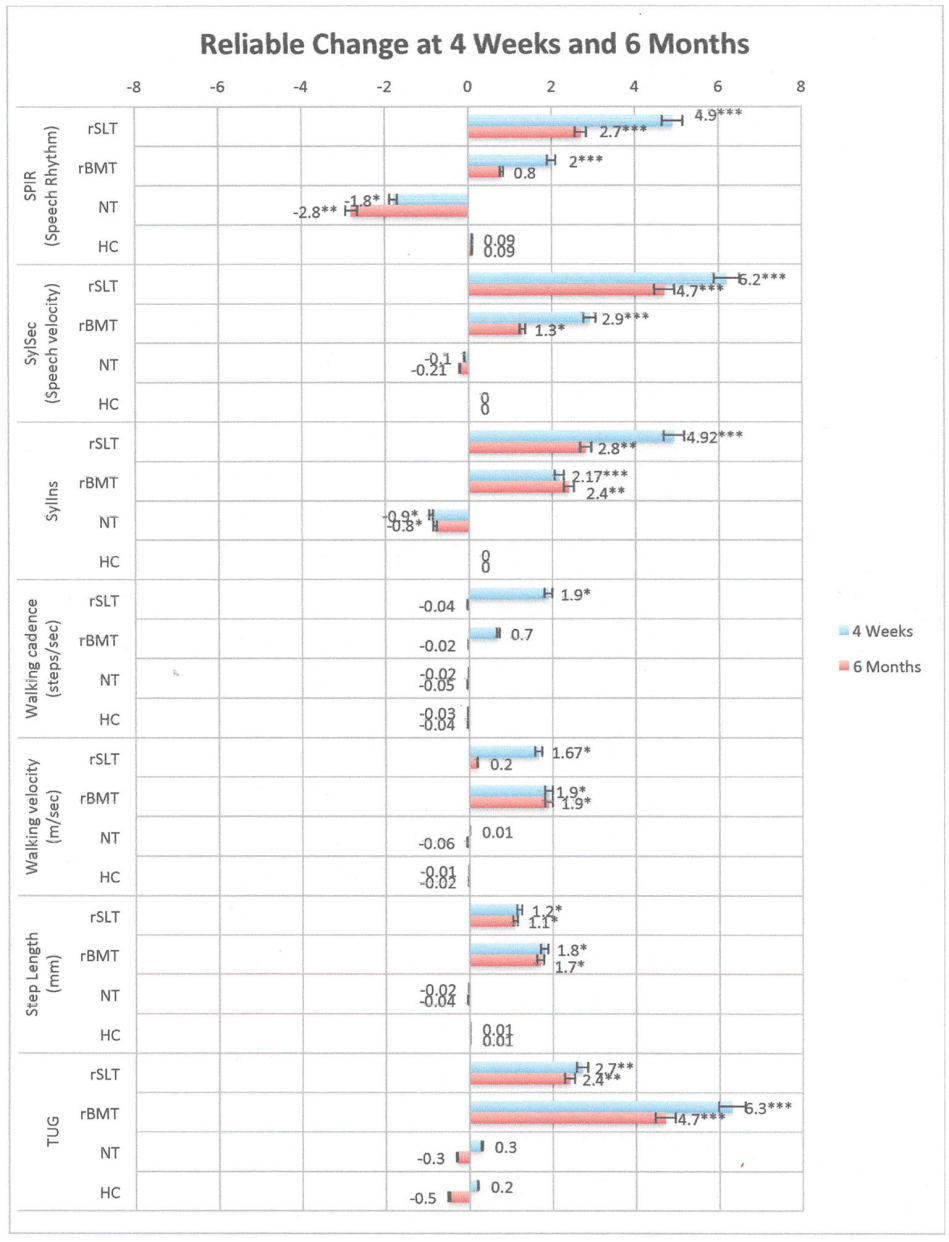
Performances of all groups across variables and time. Bars indicate Reliable change scores (Mn; SD) in performance when comparing 4 weeks to BL, and respectively, 6 months to BL. Positive scores indicate improvement, negative scores indicate a decline. Numbers marked with an asterisk are significant as follows ***p < 0.001; **p < 0.01; *p < 0.05.

Figure 3 displays all performances as RC scores across variables and time for all groups. Table 5 shows mean [range], SD, and RC scores across variables and time within groups.

**TABLE 5:** Group performances at baseline, 4 weeks, and 6 months per category as well as Reliable Change Scores and Reliable Change Index (RCI).

**Variables**		**BL**	**4 weeks**	**6 months**	**Reliable change scores^a^ (BL–4 weeks)**			**Reliable change scores^b^ (BL–6 months)**		
	**Groups**	**Mn [Range]; SD**	**Mn [Range]; SD**	**Mn [Range]; SD**	**Mn (SD)**	**RCI**	**Effect size**	**Mn (SD)**	**RCI**	**Effect size**
SPIR (speech rhythm)	rSLT	18.9 [16–22]; 1.6	12.6 [10–14]; 1.5	13.4 [10–14]; 1.2	4.9 (1.4)^***^	2.63	4.05	2.7 (1.5)^***^	1.96	4.7
	rBMT	18.6 [16–22]; 2.5	14.5 [12–16]; 1.2	17.6 [16–22]; 1.8	2 (0.8)^***^	2.01	3.5	0.8 (1.6)	3.1	0.54
	NT	17.8 [16–22]; 1.9	18.4 [16–22]; 0.92	20.1 (16–22); 1.9	−1.8 (3.1)^*^	3.24	0.27	−2.8 (2.5)^**^	3.2	1.06
	HC	10.1 [8–12]; 1.6	10 [8–12]; 1.6	10 [8–12]; 1.6	0.09 (0.1)	2.7	0.04	0.09 (0.4)	2.7	0.03
SylSec (speech velocity)	rSLT	5.3 [4.2–6.9]; 0.6	3.6 [2.8–4.8] 0.7	3.8 [2.5–5.1]; 0.8	6.2 (1.7)^***^	1.18	2.5	4.7 (3.9)^***^	1.36	1.9
	rBMT	5.3 [4.1–6.3]; 0.6	4.2 [3–5.7]; 0.9	4.8 [3.8–6.3]; 0.9	2.9 (1.2)^***^	1.59	1.2	1.3 (1.1)^*^	1.36	0.8
	NT	5.1 [4–5.9]; 0.4	5.2 [3.9–6.9]; 0.7	5.1 [4–6.12]; 0.6	−0.1 (1.2)	1.16	0.06	−0.21 (1.2)	1.04	−0.04
	HC	2.3 [1.2–3.5]; 0.6	2.3 [1.2–3.5]; 0.6	2.3 [1.2–3.5]; 0.6	0(0)	1.05	0	0(0)	1.05	0
SylINS	rSLT	8.4 [4–19]; 3.6	15.6 [7–22]; 3.7	13.3 [7–20]; 4.4	4.92 (1.2)^***^	6.24	1.9	2.8 (1.4)^***^	7.4	1.1
	rBMT	9 [4–19]; 3.9	13.1 [7–23]; 4.7	13.6 [4–20]; 4.7	2.17 (0.8)^***^	8.06	0.8	2.4 (1.5)^***^	8.03	0.9
	NT	8.6 [4–20]; 4.1	11.1 [4–15]; 3.9	11.6 [4–17]; 3.5	−0.9 (3.1)^*^	6.6	0.8	−0.8 (1.7)^*^	5.98	0.6
	HC	21.6 [14–36]; 6	21.6 [14–36]; 6	21.6 [14–36]; 6	0(0)	10.2	0	0(0)	10.3	0
TUG	rSLT	18.3 [9–43]; 10.2	12.6 [7–25]; 5.8	12.6 [6–29]; 6.9	2.7 (4.2)^**^	9.73	1.06	2.4 (2.1)^**^	12.01	0.76
	rBMT	16.3 [7–60]; 16.1	9.1 [5–20]; 2.6	11.1 [8–24]; 4.9	6.3 (8.5)^***^	5.6	0.96	4.7 (8.5)^***^	8.23	1.1
	NT	16.7 [9–25]; 4.7	15.1 [5–19]; 4.02	16.6 [8–28]; 5.3	0.3 (1.4)	6.86	0.4	−0.3 (1.2)	9.05	0.76
	HC	6.6 [4–11]; 2.4	6.6 [3–11]; 2.5	5.9 [3–10]; 2.1	0.2 (0.9)	3.4	0.3	−0.5 (2.8)	3.79	1.1
Walking cadence (steps/sec)	rSLT	1.8 [1–2.9]; 0.18	1.5 [1–2.1]; 0.34	1.8 [1–3.9]; 0.18	1.9 (4.1)^*^	0.34	−0.7	−0.04 (0.1)	0.64	0.02
	rBMT	1.7 [1.11–2.4]; 0.18	1.5 [1.11–2.78]; 0.57	1.67 [1.07–3.4]; 0.19	0.7 (1.2)	0.44	−0.3	−0.02 (0.01)	0.61	0.6
	NT	1.9 [1.07–2.5]; 0.27	1.8 [1.07–3.5]; 0.27	1.9 [1.07–3.5]; 0.18	−0.02 (0.1)	0.51	0.01	−0.05 (0.4)	0.51	0.03
	HC	1.69 [1.5–2.11]; 0.18	1.69 [1.5–2.11]; 0.18	1.69 [1.5–2.11]; 0.18	−0.03 (0.1)	0.14	−0.01	−0.04 (0.01)	0.14	0.08
Walking velocity (m/s)	rSLT	0.74 [0.4–1.05]; 0.24	0.83 [0.8–1.05]; 0.26	0.76 [0.4–1.05]; 0.48	1.67 (4.1)^*^	0.58	0.24	0.2 (0.8)	1.39	0.06
	rBMT	0.81 [0.5–1.05]; 0.27	1.15 [0.6–1.05]; 0.38	1.23 [0.5–1.05]; 0.27	1.9 (4.2)^*^	0.97	0.23	1.9 (0.2)^*^	1.36	0.02
	NT	0.8 [0.5–1.05]; 0.31	0.8 [0.5–1.05]; 0.31	0.8 [0.4–1.05]; 0.31	0.01 (0.01)	0.54	0.01	−0.06 (0.1)	1.19	0.06
	HC	1.3 [1.1–1.4]; 0.13	1.3 [1.1–1.4]; 0.13	1.3 [1.1–1.4]; 0.13	−0.01 (0.02)	0.3	−0.02	−0.02 (0.01)	0.32	0.08
Step length (mm)	rSLT	487 [400–602]; 52.1	510 [422–624]; 53.1	505 [419–618]; 54.1	1.2 (3.8)^*^	0.8	0.52	1.1 (3.8)^*^	0.8	0.52
	rBMT	485 [448–619]; 87.8	518 [452–622]; 52.1	515 [445–620]; 64.1	1.8 (3.2)^*^	0.8	0.54	1.7 (3.1)^*^	0.8	0.54
	NT	492 [400–608]; 75.4	490 [402–601]; 70.1	489 [399–598]; 87.1	−0.02 (0.1)	0.51	0.01	−0.04 (0.4)	0.51	0.03
	HC	580 [532–625]; 50.9	582 [530–628]; 50.8	581 [527–627]; 50.9	0.01 (0.02)	0.3	0.2	0.01 (0.01)	0.32	0.18

a *Reliable Change is calculated with the differences between scores at baseline and scores at 4 weeks, i.e., directly after therapy.*

b *Reliable Change is calculated with the differences between scores at baseline and scores at 6 months, i.e., after a long therapy pause.*

*Bold values are significant values as in :*

*** *p < 0.001;*

** *p < 0.01; and*

* *p < 0.05*.

#### Speech Assessments

Group comparisons revealed that although both rhythmic intervention groups significantly improved performances across variables and time, they did never reach performance levels of the HC group in any speech metric measurement (*p* < 0.001). In contrast, the NT group showed natural disease progression, because the decline in all speech variables turned significant at 6 months (*p* < 0.01).

#### Motor Assessments

Although rhythmic intervention groups revealed improvements in the TUG at 4 weeks and 6 months, they still failed to reach HC performance levels (*p* < 0.001). Further, the rSLT group showed a significant improvement in walking velocity and cadence at 4 weeks (*p* < 0.05) possibly a beneficial transfer effect. Yet, these improvements did not reach performance levels of HCs (*p* < 0.001) and vanished after 6 months. Additionally, results from the rBMT group suggested significant improvements in walking velocity at 4 weeks and 6 months (*p* < 0.05), still, these performance levels did not pattern-in with those of HCs (*p* = 0.05). Turning to step length, improvements were found significant in both intervention groups at 4 weeks and 6 months (*p* < 0.05).

On a similar note, change in performance was further noted in the rSLT and rBMT groups across sample descriptive variables specific to dysarthria (Table 1A): respiration with and without phonation (*p* ≤ *0.0*1), maximum phonation (*p* ≤ *0.0*5), motor control in articulation (i.e., movement of the lip and tongue; *p* ≤ *0.0*4), and intelligibility (*p* ≤ *0.0*5).

Turning to specific descriptive mobility parameters (Table 1B), further improvements have been observed in the one-leg balance standing test in the rBMT group (*p* < 0.05) at 4 weeks. Yet, this specific training effect disappeared at 6 months (*p* < 0.01), and their one-legged balance ability patterned in again with that of the other PD groups (*p* < 0.72). Interestingly, the HC showed a significant improvement on the tandem standing balance test at 6 months (*p* < 0.001) possibly attributable to a learning-practice effect. Turning to variables measured in the FGA-I, significant improvements were observed both intervention groups at 4 weeks in *task 1* (i.e., walking a 6 meter-long distance at a normal pace) (*p* ≤ *0.0*2) and in *task 2* (i.e., walking the 6 meter-long way with interchanging speed) (*p* ≤ 0.05). In contrast, significant deteriorations were observed in *task 5* of the FGA-I [i.e., begin to walk at a normal pace and upon hearing the acoustic signal, turn around as fast as possible (being a 180° turn) and stop, facing the other direction] in the NT group at 6 months (*p* = 0.04), possibly reflecting normal disease progression.

## Discussion

This study reveals effects and crossover effects of both intensive rhythmic interventions in patients with PD having dysrhythmic manifestations in speech and gait.

*Hypothesis 1:* Even after interventions, differences remained significant between PD and HCs across all variables. These results are in line with recent studies of locomotor (43, 58) and speech abilities (34, 38, 39, 43) in PD patients without or after treatment.

Turning first to differences reported in locomotion (43), there PD patients either underwent DBS surgery or received L-Dopa medication, or received both (43). Their walking was measured according to cadence and velocity in gait and step length. Although DBS surgery and L-Dopa medication improved walking velocity and step length, performances were still significantly different (i.e., slower walking velocity and shorter step length) to those measured in HCs. Considering speech related studies, these investigated speech differences between PD and HC participants including no interventions (39, 47, 120), or speech differences before and after an amplitude-orientated intervention [i.e., focusing on increasing speaking loudness; (34, 38)], or before and after DBS surgery and/or L-Dopa medication (43). Results suggested that firstly, PD patients have pronounced difficulties in rhythmic timing (39, 47, 120) and speech intelligibility (34, 38), and secondly, that speech abilities in PD remained outperformed by healthy subjects even after treatment (43).

These findings imply that although different treatment methods (i.e., invasive or non-invasive) are introduced in the literature and may even report temporarily beneficial effects in PD patients, these interventions are on the basis of addressing specific symptoms only and possibly temporarily ameliorating the condition. Thus, realistic expectations have to be put forward by any kind of therapy promising possible improvements, since in comparison to walking and speaking abilities in healthy subjects, disease progression cannot be slowed down, stopped or reversed (2).

*Hypothesis 2:* Our results suggest strong positive correlations between speaking rate and walking velocity, as well as between speech rhythm and walking cadence. These results are in line with previous findings (43, 48, 50, 51), therefore an underlying rhythmic dysfunction has been proposed.

*Hypothesis 3:* For rhythmic interventions, the rSLT showed improvements at 4 weeks in speech and gait (cadence and velocity), as well as in respiration, articulation, intelligibility, balance, and mobility (Tables 1A,B). Yet, at 6 months improvements were only maintained in speech rhythm and velocity (Table 5), and in respiration, articulation and intelligibility (Table 1A). A brief note regarding the short-term improvements on the one-leg balance tests. Observed changes are possibly attributable to the different standing positions involved during the therapy sessions, as depicted in Table 2. Although these were not the main focus of the method, these have an indispensable contributing factor regarding the delivery of the method. As a fact, varying standing positions requiring the patient to shift one's weight from one to the other leg and sometimes maintain these positions for a short while (i.e., during phonation exercises) additionally increased cognitive demands (i.e., concentration and awareness) in participants. Furthermore, step length significantly increased after the intensive intervention and remained significantly elongated, which may be attributed to improved balance as a coherent beneficial effect.

Turning to the rBMT group, significant improvements were once found in speech rhythm and velocity, and in spoken syllables per inspiration (SYLIns) and secondly, in walking rhythm and velocity, as well as in step length (Table 5). Further significant improvements were noted in sample descriptive variables as -for instance- in respiration, articulation, and intelligibility (Table 1A) as well as in balance (one-leg balance test) and mobility (FGA-I *tasks 1 and 2* in Table 1B). However, only improvements in walking and speaking velocity, as well as in syllables per inspiration, step length and the TUG were maintained over time. Briefly turning to the short-term improvements in the one-leg balance test (Table 1B), these may possibly be attributable to the fact that some dance trainings (i.e., at and beyond Level 3) required the rBMT participants to frequently hold a one-legged position on the dance platform during training, that is until the next training step sequences appeared on the monitor.

In contrast, performances of the untreated PD group declined over time representing the normal and expected disease progression regardless of the type of medical treatment received (i.e., DBS or L-Dopa dosage).

These results are in line with studies and reviews evaluating rhythmic motor training (52, 53, 55, 58, 59, 61–66, 91, 94, 95, 121), which reported measurements only in terms of gait and balance. Regarding rhythmic speech interventions involving singing, equivocal effects on respiration and articulation were reported (96). In addition, rhythmic speech cuing (RSC) reported improvements in intelligibility only in severe Parkinsonian speech (58).

### Clinical Implications

Apart from strong correlations between parameters in speech and gait, this study also showed that rhythmic interventions, whatever their primary therapy focus, can bring about improvement in both dysfluent domains simultaneously. These findings are novel and may support the hypothesis of a common underlying rhythmic deficit (42, 48, 51, 58). They are also in line with the consistent existing evidence (5, 9, 52, 53, 58–66, 91, 94, 95, 121) that rhythmic cues facilitate the retrieval and re-initiation of motor patterns in speech and gait having been impaired by the progression of PD.

Furthermore, as indicated by the RC scores in Table 5, our findings suggest that the effects and transfer effects of rSLT are slightly stronger than those of rBMT (except for the RC scores on the TUG). As a fact, the rSLT and the rBMT differed on the basis of *delivery mode* as well as *type of rhythmic input*.

Turning first to the *delivery mode*. The rBMT relied on an impersonal computer program to deliver the method, thus, study personnel were only present to operate the computer software and run the dance programs, protocol the scores after every dance sequence, and to assist the patient in any way if required. In contrast, the rSLT was delivered by a human being, who shows empathy and gives direct corrective feedback, both being very strong influential factors in traditional therapy, that should not be underrated. Furthermore, the therapist always adapts to the patient's ability level and the direct corrective feedback plays a key role, as it is set out to motivate and challenge the patient to achieve “more.” Yet, in the rBM-Training the computer program was set to a certain ability level, and the patient had to dance along without receiving motivational or encouraging feedback.

As for the *type of rhythmic input*, the rSLT depends on the reactivation of internally timed cues (e.g., via self-perception and monitoring of speech), while the rBMT employs external rhythmic cues (e.g., music and rhythmically appearing arrows on a monitor). An internal, naturally implemented rhythm is needed for the smooth coordination of movement sequences (9, 59). External rhythmic competency is needed for the individual to be able to perceive and respond to rhythms (122, 123) being an innate ability of humans called rhythmic entrainment (58, 124). Rhythmic entrainment can be observed in the spontaneous movement or dancing (either consciously or unconsciously) when listening to music (59), or in the adaptation of speech behavior to that of the interlocutor (89, 90, 95) or in the synchronization of steps when two people walk side by side (125–127). PD patients have pronounced difficulties with initiating internal timing (128, 129), but their ability to perceive externally timed cues seems to remain intact throughout disease progression (40, 58, 97, 130–133). External cues can also serve as a compensatory mechanism to bypass deficits of internal cueing (134).

Nonetheless, a rhythmic approach focusing on re-establishing internally timed cues would seem to be cognitively more demanding than one relying on external rhythmic cues (94, 95). By being cognitively more demanding, the rSLT is possibly more likely to induce neuroplasticity (129–132), i.e., the ability of the brain to (re-) learn, change and adapt to new demands via intensive, effortful, and goal-specific training (129). It is reasonable to suppose that the greatest benefit for patients with neurodegenerative will come from cognitively demanding and intensive training (135–138).

### Strengths of This Study

Strengths of this intervention study are (i) the crossover parallel design, (ii) the integration of reliable change and reliable change indexes in the statistical analysis, and (iii) the novel approach of comparing two different rhythmic interventions, including a no therapy group as well as healthy controls.

Advantages of a crossover design with a 3-year-long study period are that the same participants passed the intervention programs in an orderly way (Figure 2), and consequently reduced covariates, as each participant served as its own control. Naturally, the treatment order in a progressive neurodegenerative disease condition (such as Parkinson's Disease) may produce a confounding factor. To weaken this confounding factor, participants were randomly assigned to treatment groups prior to study start, and all received the same number of assessments and treatments, and all these assessments and treatments were provided parallel within timely fixed treatment tranches. Therefore, neither the natural disease progression nor the order of the treatments may have a confounding effect on the study results. Considering carry-over effects, being another potential confounding variable of crossover studies, a long enough (104–106) wash-out was integrated to weaken any carry-over effects onto following therapy tranches. Finally, all groups reached the minimum required group size of *N* = 10 per group, as calculated in the priori analyses. Taking all the above facts into account, this controlled crossover trial may be seen as optimally “balanced.”

As for reliable change (RC), it calculates the difference in performance for every individual participant (i.e., a patient's scores at pre- and post-intervention) based on firstly, how reliable the measurement is, and secondly, based on every individual participant's clinical condition at BL. By so doing, measured changes in performance become “reliable” (or *truly statistically significant*), because random fluctuation of performance due to the measurement *per se* (or “measurement error”) is filtered out. This further allows merging PD patients with DBS or no-DBS into the different groups (i.e., rSLT, rBMT, or NT), because the RC is calculated on every individual participant's clinical condition at BL. Thus, even though DBS was reported to have mixed effects on speech parameters (42, 56, 57), DBS may surely affect motor and gait functions (43, 54, 55) in PD patients. And yet, whether or not a PD participant had DBS did not affect the final outcome measure, since his clinical condition (i.e., DBS or no DBS) by being set at BL and only truly significant changes are obtained. Thus, by including the RC and the RCI in the analysis, this intervention study adheres to a high standard of examination of rehabilitative intervention trials.

Finally, the combination of two different rhythmic interventions being similar in terms of intensity and frequency (i.e., 3x per week for 45 min), but differing in terms of therapeutic approach (i.e., a speech-specific vs. a mobility-balance specific approach) allows to view the results from two different perspectives: once on effects onto primary outcome measures, and second, on transfer effects onto the opposite therapeutic focus. Therefore, this comparison enables to see whether speech and gait have a common underlying rhythmic deficit, and if so, whether these would benefit from a rhythmic intervention regardless of its primary therapy focus. Additionally, the inclusion of healthy controls as well as a no therapy PD group broadens the comparative possibilities, as effects of normal aging or natural disease progression in PD may be captured in the analyses.

### Limitations and Future Research

The rBMT group (N_rBMT_ = 11) was smaller than the others (N_rSLT_ = 16; N_NT_ = 20; N_HC_ = 17). Thus, certain comparisons cannot be taken as conclusive, since a few effect sizes remain rather marginal (η^2^ <0.2 for RC scores). Consequently, results have to be interpreted cautiously.

For speech assessments, this study focused only on audio samples read aloud, as these yield material for exact comparisons with spectral analyses, unlike audio samples of spontaneous speech, which have highly individual features and are thus difficult to compare. Nonetheless, spontaneous speech more closely reflects the communicative skills needed in everyday life.

Considering motor assessments, this study focused on the standard measurements such as the FGA, which is based on a 6 m long walk way. Yet, a longer walkway (e.g., 10 m) or a 6-min-long walk on a treadmill would have allowed to examine possible changes in cadence, velocity, and step length in more detail, since a certain variability on these accounts during locomotion may be expected. Further, the rBMT sessions were held on a spatially-limited platform, where steps were limited in terms of step length and a constant forwardly orientated direction. In contrast, a natural, real dance may have brought in variations in postural symmetry and direction. Therefore, results from this intervention cannot be generalized onto or seen as equal to other types of alternative balance-mobility rehabilitation programs.

Noteworthy is the fact that although within this cross-over intervention trial a 6-month-long washout period has been integrated between therapy tranches, carry over effects can still not be fully ruled out. Therefore, these remain as possibly existent, even if the patient's objective data may not capture carry-over effects (i.e., BL measurements administered following a completed therapy tranche and wash-out period).

Another observation is that even though rhythmic interventions generated significant effects on selective speech and gait parameters, these effects were too specific in terms of category to be captured in broader evaluations such as the UPDRS or the POMA (see Table 1B, *comparisons across time and within groups*). Furthermore, it remains questionable to how much extent these beneficial effects have had an overall effect on quality of life, since this investigation remains subject to future research.

Additionally, the effects of internal or external factors (e.g., age, level of education, sex, levodopa dosage, PD onset, depression, cognitive abilities) on the general intervention effect were not studied. If speech and gait deficits (in cadence and velocity) do indeed share a common underlying mechanism, then a common predictive influential factor should have an equally strong effect on both. This may be subject to future research.

Finally, findings of this study suggest that rhythmic cues may facilitate initiation and motor control in both speech and gait, yet, even if variables in speech and gait correlate, this is no sufficient proof for a common underlying neural mechanism. As a fact, future research should involve functional imaging techniques to study brain activation during speech and gait with and without rhythmic cues, in order to examine cortical activations as well as the effect of the type of rhythmic cue (external vs. internal) on the various brain structures involved in speech and gait.

### Conclusions

Cadence and velocity parameters deriving from speech and gait measurements seem to correlate with each other, and moreover, the two types of rhythmic intervention exert similar beneficial effects on their primary target as well as crossover effects on the other functional category (gait or speech, respectively). This new finding indicates that dysfunctions in speech and gait in PD may reflect a common underlying disturbance of rhythm. Thus, rhythmic interventions (regardless of their primary focus) may become promising rehabilitative tools because of their multifaceted effects in PD.

Furthermore, rhythmic stimulation carries effective cues to improve speech and gait parameters in PD. Finally, internally generated rhythmic cues may be more effective than externally presented ones, because they place a higher cognitive demand on the patient and are thus more likely to induce beneficial neuroplasticity.

## Data Availability Statement

The datasets presented in this study can be found in online repositories. The names of the repository/repositories and accession number(s) can be found at: Public repository (https://dataverse.harvard.edu/) For sensitive data: Department of Clinical Research (DKF; https://dkf.unibas.ch/en/about-us/contact/).

## Ethics Statement

The studies involving human participants were reviewed and approved by the Swiss Ethics Committee Northwest and Central Switzerland (Number of Ethical Approval: EKNZ 2016–01428: Evaluation of Intensive Therapy). The patients/participants provided their written informed consent to participate in this study.

## Author Contributions

AR conceived and designed the study and was responsible for its execution, wrote the manuscript, performed data analysis and data monitoring, supervised and/or provided both intervention types (i.e., speech-language therapy and dance training), contributed core ideas, and was involved in critically revising the manuscript for important intellectual content. ET contributed core ideas and was involved in critically revising the manuscript for important intellectual content. UG conceived and designed the study, was responsible for the psychiatric and psychological assessments, contributed core ideas, and was involved in critically revising the manuscript for important intellectual content. PF was responsible for its execution and data monitoring, contributed core ideas, was involved in critically revising the manuscript for important intellectual content, was principal investigator, and will act as guarantor for the data and the manuscript. All authors contributed to the article and approved the submitted version.

## Funding

This work was supported by the Bangerter-Rhyner-Stiftung (No. 8472/HEG-DSV). This institution had no further role in the design or execution of the study or in the collection, evaluation, or interpretation of data.

## Conflict of Interest

PF received grants from the Bangerter-Rhyner-Stiftung. The remaining authors declare that the research was conducted in the absence of any commercial or financial relationships that could be construed as a potential conflict of interest.

## Publisher's Note

All claims expressed in this article are solely those of the authors and do not necessarily represent those of their affiliated organizations, or those of the publisher, the editors and the reviewers. Any product that may be evaluated in this article, or claim that may be made by its manufacturer, is not guaranteed or endorsed by the publisher.
