# Supplementary material for: Evaluating a Speech-Specific and a Computerized Step-Training-Specific Rhythmic Intervention in Parkinson's Disease: A Cross-Over, Multi-Arms Parallel Study
Source: Front Rehabil Sci. 2022 Jan 14;2:783259. doi: 10.3389/fresc.2021.783259 (PMC9397933; doi:10.3389/fresc.2021.783259)
Supplement: Supplementary file 1 [file Data_Sheet_1.docx]

**Supplementary Material: Appendices**

**Appendix 1 Der Nordwind und die Sonne**

Einst stritten sich Nordwind und Sonne, wer von ihnen beiden wohl der Stärkere wäre, als ein Wanderer, der in einen warmen Mantel gehüllt war, des Weges daherkam. Sie wurden einig, dass derjenige für den Stärkeren gelten sollte, der den Wanderer zwingen würde, seinen Mantel abzunehmen. Der Nordwind blies mit aller Macht, aber je mehr er blies, desto fester hüllte sich der Wanderer in seinen Mantel ein. Endlich gab der Nordwind den Kampf auf. Nun erwärmte die Sonne die Luft mit ihren freundlichen Strahlen, und schon nach wenigen Augenblicken zog der Wanderer seinen Mantel aus. Da musste der Nordwind zugeben, dass die Sonne von ihnen beiden der Stärkere war.

By Aesop

**Appendix 2 English Translation: The North Wind and the Sun**

The North Wind and the Sun were disputing who was the stronger, when a traveller came along wrapped in a warm cloak. They agreed that the one who first succeeded in making the traveller take his cloak off should be considered the stronger one. Then the North Wind blew as hard as he could, but the more he blew, the more closely did the traveller fold his cloak around him; and at last the North Wind gave up the attempt. Then the Sun shined out warmly, and immediately the traveller took off his cloak. And so the North Wind was obliged to confess that the Sun was the stronger of the two.

By Aesop
